# Building toolkits for COPD exacerbations: lessons from the past and present

**DOI:** 10.1136/thoraxjnl-2018-213035

**Published:** 2019-07-03

**Authors:** Elizabeth Sapey, Mona Bafadhel, Charlotte Emma Bolton, Thomas Wilkinson, John R Hurst, Jennifer K Quint

**Affiliations:** 1 Birmingham Acute Care Research, Institute of Inflammation and Ageing, University of Birmingham, Birmingham, UK; 2 Respiratory Medicine Unit, Nuffield Department of Medicine, University of Oxford, Oxford, UK; 3 Respiratory Medicine, Nottingham Respiratory BRU, University of Nottingham, Nottingham, UK; 4 Clinical and Experimental Medicine, University of Southampton, Southampton, UK; 5 Academic Unit of Respiratory Medicine, UCL Medical School, London, UK; 6 Respiratory Epidemiology, Occupational Medicine and Public Health, Imperial College London, London, UK

**Keywords:** copd exacerbations, innate immunity, emphysema

## Abstract

In the nineteenth century, it was recognised that acute attacks of chronic bronchitis were harmful. 140 years later, it is clearer than ever that exacerbations of chronic obstructive pulmonary disease (ECOPD) are important events. They are associated with significant mortality, morbidity, a reduced quality of life and an increasing reliance on social care. ECOPD are common and are increasing in prevalence. Exacerbations beget exacerbations, with up to a quarter of in-patient episodes ending with readmission to hospital within 30 days. The healthcare costs are immense. Yet despite this, the tools available to diagnose and treat ECOPD are essentially unchanged, with the last new intervention (non-invasive ventilation) introduced over 25 years ago.

An ECOPD is ‘an acute worsening of respiratory symptoms that results in additional therapy’. This symptom and healthcare utility-based definition does not describe pathology and is unable to differentiate from other causes of an acute deterioration in breathlessness with or without a cough and sputum. There is limited understanding of the host immune response during an acute event and no reliable and readily available means to identify aetiology or direct treatment at the point of care (POC). Corticosteroids, short acting bronchodilators with or without antibiotics have been the mainstay of treatment for over 30 years. This is in stark contrast to many other acute presentations of chronic illness, where specific biomarkers and mechanistic understanding has revolutionised care pathways. So why has progress been so slow in ECOPD? This review examines the history of diagnosing and treating ECOPD. It suggests that to move forward, there needs to be an acceptance that not all exacerbations are alike (just as not all COPD is alike) and that clinical presentation alone cannot identify aetiology or stratify treatment.

‘Next to avoiding a fatal issue, our efforts must be directed to prevent the case going on to chronic bronchitis, especially in those who have had previous attacks’.

R Douglas Powell, London (1878)

## Introduction

An exacerbation of chronic obstructive pulmonary disease (COPD) is defined as ‘an acute worsening of respiratory symptoms that results in additional therapy’.^1^ The word exacerbation has a Latin root; stemming from the verb exacerbare meaning ‘to provoke to anger’ and the Oxford English Dictionary defines an exacerbation as ‘the process of making a problem, bad situation, or negative feeling worse’.^2^ This accurately reflects the negative impact COPD exacerbations (ECOPD) have on patient quality of life,^3^ lung function decline^4^ and mortality.^5^ In the UK, national audit data highlight the high mortality and readmission rates (and thus healthcare costs) associated with ECOPD.^6^ Exacerbations impact on patients’ quality of life^3^ and even a single exacerbation is associated with an increase in mean annual forced expiratory volume (FEV_1_) decline.^7^ The early identification, provision of appropriate treatment and subsequent prevention (or ideally, primary prevention) of exacerbations has to be a central strategy for COPD care.

We use clinical symptoms to diagnose an exacerbation of COPD, based on the triad of increased breathlessness, increased sputum volume and/ or increased sputum purulence. These criteria are essentially unchanged over the last 180 years,^8^ finessed with clinical investigations such as a chest radiograph, arterial blood gas, ECG, a full blood count and sputum culture (all available since 1872–1924).^9–13^ In stark terms, our diagnostic approach to COPD exacerbations has not fundamentally changed for almost 100 years. We have no COPD-specific biomarkers and the diagnosis is often one of exclusion. This is in contrast to many other acute presentations of chronic diseases, such as a myocardial infarction (MI) in ischaemic heart disease, where specific and sensitive diagnostic toolkits including biomarkers, imaging and interventions have revolutionised care pathways and patient outcomes. Such disparity in advancement raises the question of why COPD is so far behind other common, debilitating and progressive chronic diseases which are associated with acute flares of symptoms. Why do we not have a better diagnostic and treatment toolkit for ECOPD? Perhaps to move forward, we need to examine the past.

## Triggers of exacerbations and our ability to differentiate between them clinically

In 1878, Douglas Powell identified cold weather, upper respiratory tract infections and pollution as an important causes of (acute) bronchitis, observing that ‘dusty employments…dusty winds (and) irritating fogs’ bring on typical attacks.^14^ Today, the most important listed triggers of exacerbations of COPD include viral and/ or bacterial tracheobronchial infection^15^ and inhalation of environmental irritants.

In a study of 64 hospitalised (and thus severe) exacerbations of COPD, bacteria or viruses were identified in 78% using quantitative culture and PCR.^16^ Bacteria were present in 55% of patients, most commonly *Haemophilus influenzae*, *Streptococcus pneumoniae*, *Moraxella catarrhalis*; *Staphylococcus aureus* and *Pseudomonas aeruginosa*, in descending order of prevalence. Viruses were found in 48%, with rhinoviruses, influenza viruses, respiratory syncytial viruses, parainfluenza viruses and coronaviruses most commonly identified, again in descending order of prevalence.^16^ Viral infections are important in COPD, associated with frequent exacerbations, a higher total symptom burden at presentation and a longer period before symptom recovery,^17^ perhaps reflecting the lack of specific therapies available. Coinfection (bacterial and viral) is common (seen in 25% of severe exacerbations), associated with increased lung and systemic inflammation, longer hospitalisation and more severe lung disease.^16^ The role of air quality is of increasing interest. Short-term exposure to major air pollutants (trioxygen (O_3_), carbon monoxide, nitrogen dioxide, sulphur dioxide, particulate matter (PM)_10_ and PM_2.5_) is associated with respiratory risk but a recent systematic review concluded that these pollutants were also associated with risk of exacerbation.^18^ Other identified triggers and/or risk factors for exacerbation include discontinuation and poor adherence with medications,^19^ poor nutritional and lower socioeconomic status^20^ and dynamic hyperinflation.^21^ These causative triggers and predisposing factors have been consistently identified across the literature, but studies also highlight alternative pathologies which might account for symptoms, including thromboembolic disease and MI (identified in 16%^22^ and 8%^23^ of suspected COPD exacerbations, respectively), suggesting comorbidity is important.

The treatment of exacerbations has remained short-acting bronchodilators and corticosteroids with or without antibiotics for 30 years, almost irrespective of the underlying cause. However, with global concerns about antibiotic use and an increasing number of clinical trials of new or repurposed therapeutics given at the time of exacerbation, identifying the exacerbation trigger has never felt more relevant. Can clinical evaluation alone help identify the cause?

From the late 1990s onwards, a body of evidence supported the concept that the presence of purulent sputum at exacerbation presentation was indicative of a bacterial exacerbation (eg Stockley *et al*,^24^) and this has been used ever since as a potential biomarker for bacterial infections. However, there are concerns about the ability of patients to self-report sputum colour without training or a colour chart to refer to Daniels *et al* 25 and in some studies, sputum colour could not differentiate between a viral or bacterial aetiology.^16^ A landmark study in 2011^26^ suggested that exacerbations could be stratified using inflammatory profile. Here, 55% of exacerbations were associated with bacteria, 29% with a virus, most commonly *rhinovirus,* 28% with a significant sputum eosinophilia and 14% with no significant inflammation (termed pauci-inflammatory). Of note, these groupings did not reflect differences in symptom burden, clinical presentation or sputum colour, which could not differentiate between causes,^26^ meaning clinicians could not predict what the cause or inflammatory profile of the exacerbation was using standard clinical evaluation alone. This has important but perhaps predictable implications for clinical practice. Moving forward, as with other acute presentations of chronic disease, we should not rely on clinical symptoms, signs or non-specific investigations to direct a stratified approach to exacerbation treatment. Given COPD is an inflammatory disease, the immune response may provide aetiological insight.

## The host response to exacerbations

In 1958 Engel hypothesised that the structural lung damage described in chronic bronchitis and emphysema might be caused by repeated infections, with multiple acute insults leading to long-term lung damage.^27^ In 1968, Morgan suggested that there were differences in the acute and chronic inflammation seen in the bronchial tree and surmised that these differences may influence patient outcome and require different treatments.^28^ Fifty years on from this observation, how far has our understanding of the inflammatory basis of acute exacerbations of COPD progressed?

There is a substantial and convincing body of evidence that airway inflammation is prevalent in stable COPD and is fundamental to its pathogenesis with studies suggesting relationships with disease severity and inflammatory burden.^29^ However, pulmonary inflammation varies greatly between individuals and within individuals with COPD even when clinically stable^30^ and this heterogeneity has proven challenging in biomarker evaluation or inflammation-targeted therapeutic intervention.

**Figure 1:**
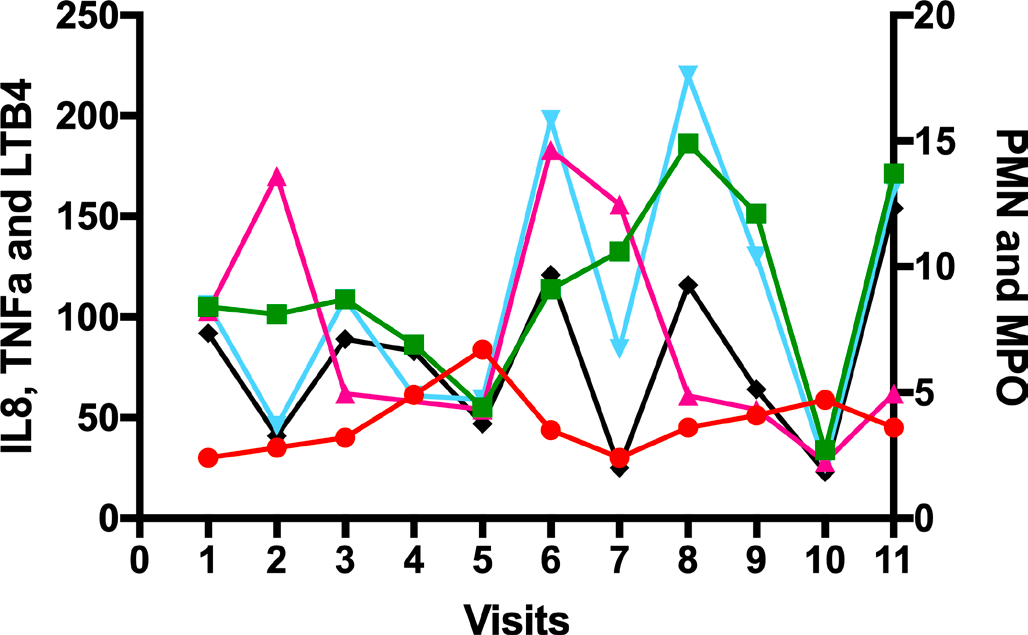
The variability of inflammation in sputum in one patient with COPD. A spontaneous sputum sample was collected over 4 hours post waking and following mouth rinsing procedures daily for 5 days (visits 1–5), and then twice weekly for 3 further weeks (visits 6,7; 8,9; 10,11) in a patient with moderate severity COPD in the stable state who had been an ex-smoker for 17 years. A differential cell count was performed and cytokines were measured in sputum sol phase. Each marker is the concentration of that mediator on the visit day. Neutrophil (PMN) 10^6^/ml (red circles), myeloperoxidase (MPO) mg/ml (green square); TNFα pM (cerise triangle): IL8 nM (cyan triangle); LTB4 nM (black diamond). adapted from Sapey *et al*.^29^ COPD, chronic obstructive pulmonary disease; TNF, tumour necrosis factor.

Figure 1 provides an example of the variability of the inflammatory profile of spontaneous sputum inflammation day to day in one patient with COPD. As shown, although some mediators share a common pattern of change (if one mediator is up, others are up and vice versa), not all do (eg, on visit 2, tumour necrosis factor (TNF)α has increased, but leukotriene B4 (LTB4) and interleukin-8 (IL8) have decreased) and this suggests that the variability in mediators does not only reflect dilution of the sample, but true variability in inflammatory pattern.

**Figure 2:**
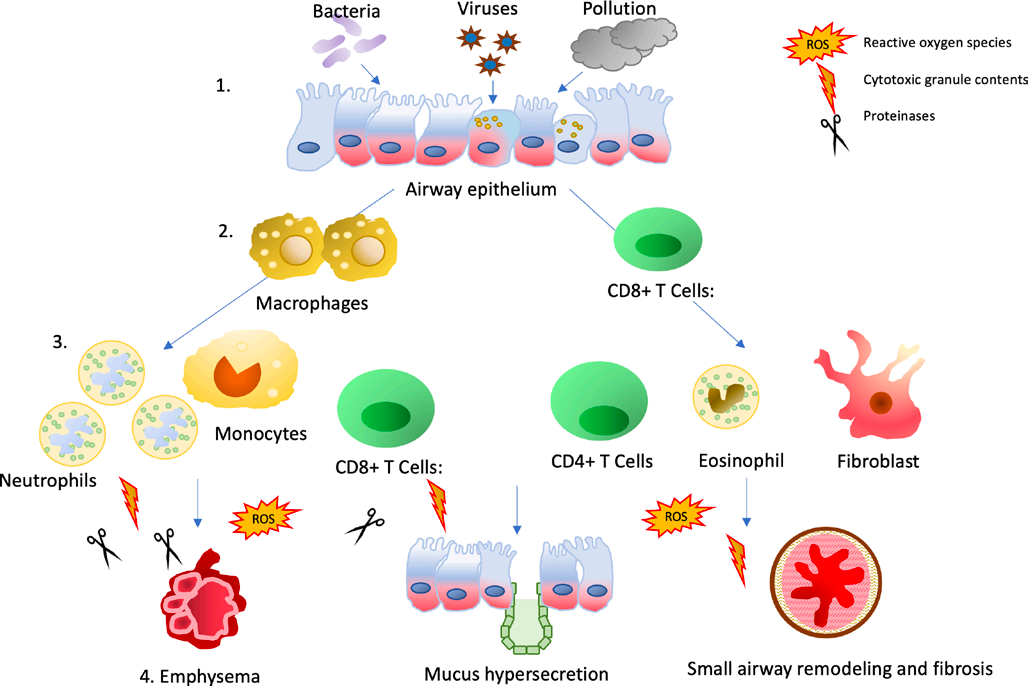
The inflammatory cascade in COPD exacerbations. A summary of inflammatory and cellular interactions linking exacerbations to the chronic inflammation of COPD. 1. The precipitating event (which could be a bacterial or viral infection or an environmental trigger causes inflammation of the airway epithelium). 2. Activation of the resident immune cells including macrophages and T cells. 3. Where local host defences are overwhelmed, non-resident immune cells, predominantly neutrophils, but also T cells, B-cells and eosinophils are recruited into the lung tissue, following chemokines secreted by epithelial and endothelial cells and resident immune cells. Fibroblasts may be activated by growth factor releases from macrophages and epithelial cells. 4. Recruited and resident immune cells are able to release cytotoxic granular contents, reactive oxygen species and proteinases into the tissue and these have been causally associated with the development of mucus secretion, but also emphysema and small airways remodelling, leading to progression of the underlying COPD.

There appears to be a further amplification of inflammation during exacerbation in many (but not all) patients. Once an insult (bacterial, viral or environmental) sufficiently activates the resident immune cells of the airways, it appears to trigger a cascade of inflammatory mediators.^31^ This in turn recruits a wave of activated immune cells to the airways, which are predominantly neutrophils^16^ but also include eosinophils, monocytes and CD8 +T cells^32^ ^33^ and these cells have the potential to cause significant disruption and damage when they enter tissue *en masse*. For example, activated neutrophils release proteinases during migration through complex tissues, degranulation, frustrated phagocytosis and Neutrophil extracellular traps (NET) formation.^34^ The concentration of proteinases initially far exceed and thus overwhelm their inhibitors leading to degradation of structural lung proteins including elastin and collagen causing bystander tissue damage and cleavage of enzymes, cytokines, receptors and opsonins including components of the complement cascade and immunoglobulins.^35 36^ While tissue damage is heightened in exacerbations of COPD, tissue repair is blunted, effecting the structural integrity of the airways.^37^ This inflammatory cascade also results in systemic inflammation, with increases in acute phase proteins such as fibrinogen and C reactive protein (CRP). Relationships have been described between the degree of pulmonary and systemic inflammation in some studies,^38^ a potential link between the multimorbid diseases associated with COPD and COPD exacerbations (see later). These processes are illustrated in figure 2.

COPD severity (definable by a number of measures, but commonly by FEV in one second (FEV_1_) at one timepoint) is not synonymous with COPD activity (the trajectory of lung function decline or exacerbation frequency). Some patients have mild COPD by FEV_1_ which is rapidly progressing or with frequent exacerbations and vice versa. While hypothetically attractive, studies have not consistently linked disease activity (such as exacerbation frequency) to the presence of increased airways inflammation in the stable state.^39^ However, the presence of potentially pathogenic bacteria on sputum culture is associated with exacerbation frequency^40^ and there is a clear relationship with bacteria and inflammation^39^ which supports the concept of inflammatory burden increasing the susceptibility to exacerbations. It is likely that some studies have been underpowered to assess differences in inflammation or have failed to include patients with high exacerbation frequencies, which is understandable given the challenges of recruiting these unstable patients to research studies.

There is an association between inflammation and exacerbation outcome. Symptom resolution corresponds to abating inflammation and continuation of symptoms or recurrence of exacerbation corresponds to sustained inflammation,^41^ suggesting a causal relationship between inflammatory load and host experience. While it is attractive to assume that all exacerbations of a certain aetiology might share the same inflammatory profile and burden, the complexity of host, environment and exacerbation trigger interactions within COPD are likely to produce patterns with greater subtlety than that. However, just as with stable COPD,^42^ within COPD exacerbations there might be phenotypes or ‘treatable traits’ which could help focus therapeutic choices.

Immune cell function might provide mechanistic insight. It has been proposed that some frequently exacerbating COPD patients might experience a ‘triple innate immune system hit’ which could increase their susceptibility to bacterial exacerbations. First, the frequent exacerbator phenotype has been associated with a reduced ability of airway macrophages to phagocytose bacteria.^43^ Theoretically, this would lead to increased neutrophil recruitment and in this group neutrophilic inflammation is commonly described.^31^ Second, studies suggest the accuracy of neutrophil targeting is impaired in COPD and associated with heightened bystander tissue damage.^44^ Third, airway macrophages and monocytes from the frequent exacerbator phenotype are less able to clear dead and dying neutrophils (and eosinophils^45^ via efferocytosis,^46^ resulting in cell necrosis and localised inflammation and tissue damage). Neutrophilic inflammation is corticosteroid resistant in COPD but promisingly, studies have identified potential therapeutic targets to improve impaired cellular functions. NrF2 activators increase macrophage phagocytosis^46^ and PI3K inhibitors have been shown to increase neutrophil migratory accuracy in vitro as well as reducing inflammation^47^ with PI3K inhibitors under assessment in early phase studies as a potential therapy during COPD exacerbations.

Due to advancements initially in asthma care, trials of therapies in those COPD patients with an eosinophil signal are well underway (with 70 studies currently listed on the clinicaltrials.gov website).^48^ Results to date suggest that this trait is associated with a good treatment response to oral steroids at exacerbation^49^ and inhaled steroids in the stable state,^50^ with studies of specific antieosinophil therapies (including mepolizumab) showing promise in selected patient groups.^51^ Furthermore, studies of community-treated exacerbation suggest that there is no advantage in treating adults without an eosinophil signal with oral prednisolone, as this provides no symptomatic benefit and an increase risk of harm.^49^ In hospitalised ECOPD, studies suggest that oral corticosteroids and shorter courses appear adequate, with no benefit using high-dose intravenous therapy.^52^ Excessive use of oral corticosteroids is associated with harm, which is especially clear in studies of patients on long-term maintenance^53^ but also potentially raises concerns about uncontrolled and/or unsupported use of ‘rescue packs’. Of note, a recent Cochrane review concluded that there was no evidence of benefit from self-management interventions (including rescue packs) to reduce all‐cause hospital admission, all‐cause hospitalisation days, emergency department visits, general practitioner visits, dyspnoea scores, the number of COPD exacerbations or all‐cause mortality^54^ although more research was needed. However, the provision of a rescue pack for patients with exacerbations remains a recommendation from the National Institute for Health and Care Excellence in the revised guideline published in December 2018 (based on expert opinion).^55^

These studies begin to highlight that there are different types of COPD exacerbations, with different responses to treatment and that a ‘one size fits all’ approach for both treatment and prevention is overly simplistic. To further advance inflammation-based treatments, a toolkit is needed to match exacerbation aetiology with host response and therefore treatment. In other words, we need to phenotype exacerbations.

## Phenotyping exacerbations

The value of phenotyping exacerbations of COPD is to derive patterns for treatment response or to enhance our understanding of underlying mechanisms. A frequently used, yet rudimentary classification of an exacerbation phenotype is the categorisation as ‘infective’ or ‘non-infective’ exacerbations of COPD.^56^ This is commonly used to direct treatment with antibiotics and systemic corticosteroids, respectively^57^ but does not inform underlying mechanisms, likely treatment response or if the exacerbation severity and outcomes are the same. Recent advances which exploit developments in biomarker identification, mediator discovery and molecular diagnostics,^58 59^ for example in microbial detection, have furthered our understanding of the exacerbation event.

### Biological exacerbation phenotypes: systemic biomarkers

There has been great interest in studying systemic plasma samples in COPD to provide insight into the pathogenesis of exacerbations. One such study included 90 exacerbating patients (unselected, of any aetiology), assessing 36 preselected inflammatory mediators. Of these, CRP, interleukin-6, myeloid-progenitor inhibitory factor 1, pulmonary and activation-regulated chemokine, adiponectin and soluble intracellular adhesion molecule-1 were significantly elevated at the exacerbation event. However, no plasma mediator alone provided a robust predictive tool for diagnosing an exacerbation event. CRP combined with a major symptom (dyspnoea, sputum purulence or sputum volume) improved diagnostic accuracy but no mediator/symptom combination predicted clinical severity or recovery.^60^ This result may reflect that the study included ‘all comers’ with an exacerbation (all aetiologies) and therefore might have been underpowered to find predictive biomarkers if the biomarkers varied by aetiology or host response. To address this, further studies utilised differing approaches to identify phenotypes of exacerbations.

### Biological exacerbation phenotypes: human rhinovirus biomarkers

The first attempts to investigate biomarkers in virus-associated exacerbations as a specific phenotype were made from the East London Cohort.^61^ In this study, human rhinovirus (HRV) infection was examined in healthy controls and COPD patients at stable state and during exacerbation.^61^ Baseline CXCL10 (interferon gamma inducible protein 10) was higher in COPD than controls, but at exacerbation, there was an increase in serum CXCL10 in HRV positive exacerbations, correlating with sputum HRV virus load, and no increase in HRV negative exacerbations. A combination of ‘cold’ symptoms and serum CXCL10 at exacerbation was associated with a ROC of 0.82 in predicting an HRV-associated exacerbation of COPD.

### Biological exacerbation phenotypes: independent biological clusters

The studies described so far tested preformed hypotheses to identify associations between inflammatory profiles and exacerbation. In the first study of its kind, the BEAT-COPD study employed cluster analysis using mediators sampled from the airways to determine biologically distinct exacerbation groups.^62^ Four biological exacerbation phenotypes were described, mapping on to inflammation, independent of each other but clinically indistinguishable. Sputum interleukin 1β (IL1β) was found to be most sensitive for bacteria-associated exacerbations (proinflammatory cluster, Receiver Operating Characteristic curve (ROC) 0.89), serum CXCL10 was (again) most sensitive for virus-associated exacerbations (Th1 cluster, ROC 0.76) and peripheral blood eosinophils (Th2 cluster, ROC 0.85) was the most sensitive for sputum eosinophilic-associated exacerbations. An independent validation cohort of 89 subjects confirmed that sputum IL1β, serum CXCL10 and peripheral blood eosinophils continued to show predictive power for identifying phenotypes, with ROC of 0.73 (0.61–0.85), 0.65 (0.52–0.78) and 0.95 (0.87–1.00), respectively.

These studies highlight four potential exacerbation phenotypes which might provide robust treatment pathways in time. 1. Bacterial in origin, IL-1β as a biomarker, neutrophilic inflammation. 2. Viral in origin, with CXCL10 as a biomarker. 3. Eosinophillic in origin and as a biomarker. 4 Pauci-inflammatory. These appear to be biologically different even when clinically indistinguishable. However, while our understanding of each of these phenotypes needs to be improved, we understand very little at all about the so-called pauci-inflammatory exacerbation. Indeed, it is unclear whether this represents COPD at all or the acute presentation of a related comorbidity which may also cause or exacerbate breathlessness and a cough.

## Comorbidities and COPD exacerbations

**Figure 3:**
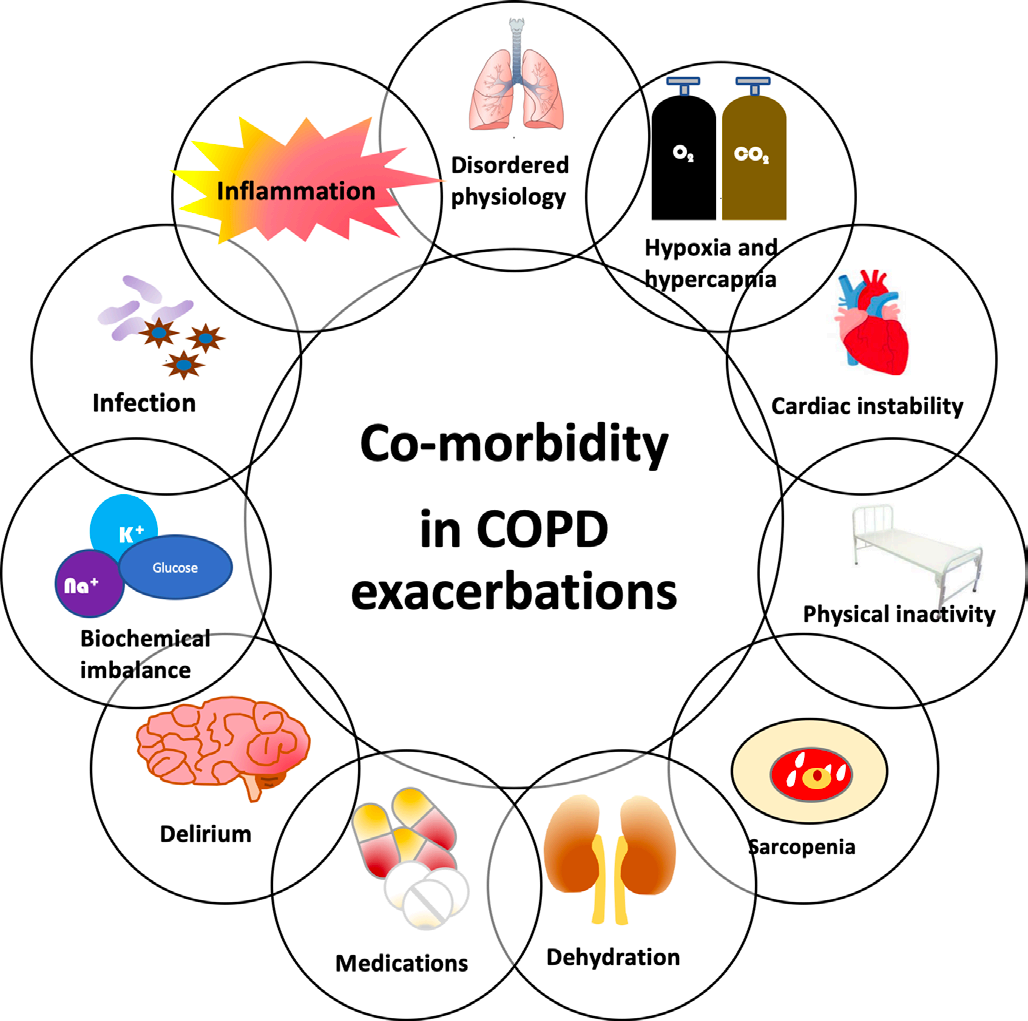
Comorbidity and COPD exacerbations. There are many stressors during COPD exacerbations which can predispose to or exacerbate comorbidities and the multimorbidity patients experience. This figure is a schematic of some of these factors, but is not exhaustive and each stressor can influence the other, irrespective of placement in the figure. Stressor include the direct effects of infection and inflammation, dyshomeostasis including hypo and hypernatraemia, kalaemia and glycaemia, hypoxia and hypercapania. Organ dysfunction is common, especially of cardiac and renal origin. Patients are placed on short courses of oral corticosteroids and physical activity is reduced (and can equate to bed rest in some patients), both contributing to sarcopenia and osteopenia. COPD, chronic obstructive pulmonary disease.

The recognition and gravitas of comorbidities in COPD has built over the last decade or more. Whether the presence of comorbidities is based on self-report^63^ or systematically sought,^64^ they are common and affect mortality.^65^ Exacerbations represent a period with multiple insults to both the lung and systemically. Such insults include the aetiological factor itself (pathogen or environmental), lung physiological changes and additional work of breathing, hypoxia, periods of inactivity (which can effectively be prolonged periods of ‘bed rest’ during an in-patient admission), with a study suggesting that an acute medical admission is associated with a median step count of 626 per day (IQR 266–1403),^66^ dehydration, malnutrition, the therapies prescribed and their side effects (eg, oral corticosteroids and hyperglycaemia and antibiotics and gastrointestinal disturbance) and then the sequelae of these factors including systemic inflammation, hypo and hypernatraemia/kalaemia and altered sympathetic drive. Figure 3 summarises the complex relationships between comorbidity and exacerbations in COPD. There is a significant and complex interplay between the exacerbation and the comorbid condition including the impact of comorbidities on the exacerbation itself; how an exacerbation contributes to comorbid disease; the prognostic role of comorbid disease and the subclinical presentation of a comorbid condition at the time of an exacerbation. Cardiovascular disease highlights the interplay and is the most studied comorbidity in this context.

Patients presenting to hospital with a COPD exacerbation have a host of comorbid conditions^67^ and the presence of a comorbid condition and the systemic manifestation of that comorbidity increases the duration of an exacerbation. Coexistent ischaemic heart disease leads to far greater number of symptomdays per year,^68^ while an increased blood glucose in hospitalised patients leads to a longer stay and is associated with a higher risk of death.^69^ MI is more likely in the period following presentation with an exacerbation^70–72^ and there is evidence of increase platelet aggregation, increased arterial stiffness as well as myocardial injury as evidenced by cardiac biomarkers at the time of a COPD exacerbation.^73 74^ The fact that comorbid disease may present subclinically at the time of the exacerbation is also important to consider, be it as a differential or as a further contributing factor to the symptoms and challenges of managing the condition. In a prospective case series, one in 12 patients presenting to hospital with an exacerbation of COPD had criteria that would meet diagnosis of a MI.^23^ Impaired cognitive function is evident, if assessed, in a large proportion of patients at the time of discharge from a hospitalised exacerbation, with no evidence of recovery 3 months later.^75^

Prognostically, comorbidities present a greater risk of hospitalisation,^76^ particularly in the presence of lower lung function, as well as increased all-cause readmissions related to multimorbidity and older age.^77^ In the ECLIPSE study (Evaluation of COPD Longitudinally to Identify Predictive Surrogate End-points), the best predictor of exacerbations was a former history of them. In addition, however, a history of reflux and heartburn was a further independent factor.^78^ The presence of acute kidney injury^79^ and lower limb muscle cross-sectional area at the time of exacerbation requiring hospitalisation are both prognostic of death.^80^ The prognostic COPD exacerbation score such as the validated DECAF score^81^ (“Dyspnoea, Eosinopenia, Consilidation, Acidaemia and atrial Fibrillation” score predicting in-patient mortality) and the PEARL score (“Previous admissions, eMRCD score, Age, Right-sided heart failure and Left sided heart failure” score predicting 90-day readmission and mortality)^82^ include cardiac comorbidity in their calculations. Patients deemed as frequent exacerbators are more likely to be depressed^83 84^ or have coexistent cardiovascular disease or osteoporosis.^84^

It is unclear if some events labelled exacerbations are actually a presentation of a comorbid condition (and studies suggest that clinicians are less likely to diagnose MI or Pulmonary Embolus (PE) if there is a concomitant diagnosis of COPD,^85 86^) perhaps the so-called pauci-inflammatory exacerbations, or whether the comorbidity is exacerbating the COPD.

There remains a role for more timely identification of comorbid disease and addressing the contributing factors. The role of systematic identification of certain comorbidities and of preventative strategies, both pharmacologically and lifestyle-based are topics for ongoing discussion and research.^87 88^ In the meantime, opportunity exists to ensure optimal treatment for those with identified comorbid disease, such as ensuring beta-blockers are prescribed in those who meet the criteria or that hyperglycaemia or hyperlipidaemia are adequately addressed.^89^

## Exacerbation treatment

Despite a greater understanding of the biology and complexity of COPD exacerbations, this has not (yet) translated into novel therapies to treat exacerbations. There has been no new intervention to treat COPD exacerbations since the widespread adoption of non-invasive ventilation to treat exacerbations with hypercapnoeic respiratory failure in the early 1990s. From the first introduction of guidelines such as GOLD in 1997, the therapy for an exacerbation is unchanged. As described below, despite being commonly used, there remain significant research knowledge gaps in determining which exacerbations do and do not require treatments with antibiotics and corticosteroids.

Systemic corticosteroids were first used in rheumatological disease during the late 1940s. Despite evidence in the late 1980s that many hospitalised patients were being treated with systemic corticosteroids,^90^ it was only at the turn of the millennium that small randomised clinical trails (RCTs)first documented clinical efficacy, suggesting benefit on lung function and outcomes such as length of hospital stay.^91^ Around the same time, the first small outpatient trials of steroids at exacerbation reported,^92^ with modest benefits confirmed in a larger 2003 RCT.^93^ Later it was defined that short course (5 days) treatment was as effective as longer 14 day courses, and without the need to taper dose.^94^ With a greater emphasis on exacerbation phenotyping, more recent studies have documented the ability to safely withhold steroids in exacerbations without an eosinophil signal.^49^ However, the practicality of achieving this at point-of-care, and the optimal blood eosinophil cut-off to guide steroid therapy remain to be determined, and there are ongoing trials in the area. Given the toxicity associated with repeated courses of corticosteroids, the need for effective novel anti-inflammatory agents is also great. Disappointingly, there is no evidence of benefit with the anti-TNF agent entanercept^95^ or roflumilast,^96^ for example.

Anthonisen’s 1987 RCT demonstrated the superiority of antibiotics over placebo in exacerbations presenting with at least two of the three cardinal symptoms of increased breathlessness, sputum volume and sputum purulence.^97^ Importantly, this had been conducted in patients with COPD, rather than just those with chronic bronchitis. However, the placebo response rate was high, likely reflecting viral pathogens as a common cause of exacerbation, and more recent studies have not shown a benefit of antibiotics in other outcomes such as prolonging the time to next exacerbation.^98^ Biomarkers such as sputum colour and procalcitonin^99^ have been suggested as strategies to better guide antibiotic therapy, but there remains unmet need to better define which exacerbations do and do not benefit from antibiotic therapy. It is also notable that there are no effective interventions to treat (or prevent) rhinovirus infections, thought to be the single the most common cause of a COPD exacerbation.

Salbutamol has been available since the late 1960s, with ipratropium following in the 1970s. These replaced the non-selective β adrenoreceptor agonist isoprenaline. There are no good data on long-acting bronchodilator drugs at the time of exacerbation.

The 1980s audit referred to above^90^ highlighted the widespread use of theophyllines (in 48% of patients), and use of respiratory stimulants such as doxapram in the management of hospitalised exacerbations. Use of theophylline has reduced, while respiratory stimulants have been replaced by non-invasive ventilation for the management of hypercapnoeic respiratory failure in the respiratory ward environment, following initial studies in the early 1990s^100^.

Models of care have changed, with the recognition that earlier access to treatment for exacerbations can be associated with faster recovery and reduced risk of hospital admission.^101^ However, the risks and benefits of patient-held rescue packs remain to be definitely established.^54^

Research to develop new interventions at exacerbation of COPD is hampered by robust outcome measures to assess exacerbation recovery. Changes in lung function are not patient centred, and changes in symptoms scores not validated. ‘Clinical recovery’ and ‘treatment failure’ are subjective constructs, while studies have also examined effects of exacerbation treatment on the time to the next event given that exacerbations cluster in time, with a high-risk period for a second event in the period following recovery from a first.^102^

We have at least made progress in prevention of exacerbations, though even when used optimally there seems to be a ceiling of reduction at around 25%. Effective interventions (outlined in table 1), alone and in combination, include non-pharmacological approaches such as pulmonary rehabilitation, and pharmacological approaches the mainstay of which remains long-acting bronchodilators with or without inhaled corticosteroids and, in selected cases, prophylactic antibiotics. For patients remaining hypercapnic following a hospitalised exacerbation, domiciliary non-invasive ventilation significantly reduces the risk of rehospitalisation (with an absolute risk reduction of 17% in a recent landmark study).^103^ Similar to strategies to better target exacerbation treatment, there is also now emerging evidence on how better to target exacerbation prevention interventions, including the optimal use of inhaled corticosteroids. Thus, while exacerbation prevention strategies are incompletely effective, the challenge here is rather selecting the right combination of interventions for the right patient at the right time, rather than the absence of effective prevention strategies.

**Table 1:** Interventions that have been demonstrated to reduce the risk of exacerbation and/or hospitalisation in patients with COPD, alone or in combination^106^

Non-pharmacological	Pharmocological
Influenza vaccination	Inhaled corticosteroid
Pneumococcal vaccination	Long acting beta agonists
Pulmonary rehabilitation	Long acting antimuscarinics
Volume reduction interventions	Long-term macrolide antibiotics
Domiciliary non-invasive ventilation	Mucolytic-antioxidants
	PDE4 inhibitors
	Anti-IL5

Not all interventions are effective in all patients and a personalised approach is mandatory.

COPD, chronic obstructive pulmonary disease.

## Conclusions

In 1878, R Douglas Powell advised that our management aims should be to save life and prevent further episodes of then acute and chronic bronchitis, now COPD. We still have a long way to go to achieve this. Exacerbations of COPD are still associated with significant mortality, morbidity, readmission and poor life quality. There have been no real advancements in routine care since the 1990s. There is considerable unmet need for novel strategies to identify, treat and prevent exacerbations, and a pressing need to better use existing therapies. This remains a major challenge. How do we move forward?

COPD as a disease concept has always raised the question of ‘lumping or splitting’; is this one disease or many?^104^ Innovation in asthma care has provided a path which perhaps COPD should follow. Asthma phenotypes and now endotypes^105^ provide clinically blurred but biologically distinct clusters with an emerging arsenal of treatments for those with the most difficult to manage symptoms. The concept of ‘treatable traits’ has gained considerable momentum in stable COPD,^42^ and perhaps now the same concept should be tested further in exacerbations. We are beginning to see some differences in biological signals across exacerbation aetiologies and host responses. To build on this, we need to continue with more stratified studies of ECOPD, learning from the fruitful experience of focusing on those with an eosinophilic signal, but this time using POC testing to characterise and test treatments in (eg) viral or pauci-inflammatory exacerbations. This will provide more information about aetiology, but to personalise treatment, this must be incorporated into a holistic understanding of the impact of the hosts comorbidity and immune responses.

From these data, we could build our ECOPD toolkit, which we hypothesise might include POC identification of bacterial or viral pathogen (ensuring that the correct antibacterial or viral therapy is used and thus reducing redundant therapy), blood biomarkers to identify or exclude an eosinophil (corticosteroid use or avoidance) or cardiac (acute coronary syndrome, heart failure) or neutrophilic treatment pathway and a measure of acuity and respiratory compromise. By exploring these ideas, we may be able to introduce a stratified approach to treatment and prevention, which might, finally, really impact on these debilitating and costly events, to the benefit of our patients.
